# An Erythrocyte Vesicle Protein Exported by the Malaria Parasite Promotes Tubovesicular Lipid Import from the Host Cell Surface

**DOI:** 10.1371/journal.ppat.1000118

**Published:** 2008-08-08

**Authors:** Pamela A. Tamez, Souvik Bhattacharjee, Christiaan van Ooij, N. Luisa Hiller, Manuel Llinás, Bharath Balu, John H. Adams, Kasturi Haldar

**Affiliations:** 1 Department of Pathology, Feinberg School of Medicine, Northwestern University Chicago, Illinois, United States of America; 2 Department of Microbiology-Immunology, Feinberg School of Medicine, Northwestern University Chicago, Illinois, United States of America; 3 Allegheny General Hospital, Allegheny-Singer Research Institute, Center for Genomic Sciences, Pittsburgh, Pennsylvania, United States of America; 4 Department of Molecular Biology, Lewis-Sigler Institute for Integrative Genomics, Princeton University, Princeton, New Jersey, United States of America; 5 Department of Biological Sciences, University of Notre Dame, Notre Dame, Indiana, United States of America; Case Western Reserve University, United States of America

## Abstract

*Plasmodium falciparum* is the protozoan parasite that causes the most virulent of human malarias. The blood stage parasites export several hundred proteins into their host erythrocyte that underlie modifications linked to major pathologies of the disease and parasite survival in the blood. Unfortunately, most are ‘hypothetical’ proteins of unknown function, and those that are essential for parasitization of the erythrocyte cannot be ‘knocked out’. Here, we combined bioinformatics and genome-wide expression analyses with a new series of transgenic and cellular assays to show for the first time in malaria parasites that microarray read out from a chemical perturbation can have predictive value. We thereby identified and characterized an exported *P. falciparum* protein resident in a new vesicular compartment induced by the parasite in the erythrocyte. This protein, named Erythrocyte Vesicle Protein 1 (EVP1), shows novel dynamics of distribution in the parasite and intraerythrocytic membranes. Evidence is presented that its expression results in a change in TVN-mediated lipid import at the host membrane and that it is required for intracellular parasite growth, but not invasion. This exported protein appears to be needed for the maintenance of an essential tubovesicular nutrient import pathway induced by the pathogen in the host cell. Our approach may be generalized to the analysis of hundreds of ‘hypothetical’ *P. falciparum* proteins to understand their role in parasite entry and/or growth in erythrocytes as well as phenotypic contributions to either antigen export or tubovesicular import. By functionally validating these unknowns, one may identify new targets in host–microbial interactions for prophylaxis against this major human pathogen.

## Author Summary

*Plasmodium falciparum*, the most virulent form of human malaria, causes disease when it invades a red blood cell. It sends proteins beyond its borders into the host, changing the red cell to make it a suitable environment to live in and to interact with the host immune system. Recent findings have predicted that hundreds of parasite proteins are released into the host red cell. However, most of these proteins have no known function. One major challenge is to understand what role these proteins play in parasite growth in order to design drugs or vaccines that block protein function. In this study, we designed a strategy to characterize ‘hypothetical’ proteins and use one as an example to illustrate the principle. We show that this protein resides within a novel compartment within the red cell and changes lipid transport at the host surface to stabilize a major nutrient pathway formed within the human cell. In principle, this strategy is applicable in determining the functions of other parasite genes involved in pathogen–host interactions.

## Introduction

Blood stage infection by *Plasmodium falciparum* causes all of the disease symptoms and pathologies associated with malaria [1],[2] and begins when the extracellular ‘merozoite’ stage invades the mature erythrocyte. The newly formed intracellular ‘ring’ stage parasite is surrounded by a parasitophorous vacuolar membrane (PVM). As ring parasites mature to the ‘trophozoite’ stage, a tubovesicular network (TVN) buds as a series of interconnected vesicles from the PVM into the host erythrocyte to support import of nutrients as well raft proteins and lipids from the erythrocyte membrane [3],[4]. In addition to creating an import pathway and modifying permeation properties of the host membrane by a non-selective ion channel [5], the parasite alters its host in other ways. Increasing adhesiveness of the infected erythrocyte to endothelial cells allows the parasite to escape splenic destruction [6]. Stabilizing the erythrocyte cytoskeleton also protects against the damaging effects of febrile temperatures [7].

Several hundred parasite proteins predicted to be exported to the erythrocyte [8]–[10] presumably underlie the molecular basis of erythrocyte remodeling in order to make this host cell a suitable environment for intracellular parasite growth. Most are ascribed as ‘hypothetical’ proteins, and since genetic manipulation of this parasite remains limited, their role in infection is poorly understood. Nonetheless, non-essential proteins involved in antigen export to the erythrocyte as well as stabilization/destabilization of the erythrocyte cytoskeleton [7],[11] have been increasingly amenable to study. Prevalent molecular and genetic analyses have thus assessed their contribution to antigen export and/or function at the host membrane [12],[13]. In contrast very little is understood at the molecular/genetic level about the biogenesis of the TVN since genes underlying it are expected to be essential for infection and cannot be knocked out.

At the time of its discovery, the biosynthesis of sphingomyelin was recognized to be an import feature of the TVN. Inhibiting a parasite sphingomyelin synthase activity exported to the erythrocyte [14] with sphingolipid analogues (such as *dl-threo*-1-phenyl-2-palmitoyl-3-morpholino-1-propanol; PPMP) [4] blocked formation of TVN tubules and their import functions. By blocking the TVN, PPMP does not immediately kill the parasite but rather arrests its development, an effect that can be completely reversed by washing out the drug even after 24 h. To facilitate the identification of genes that regulate the TVN, we examined the global transcriptional profile of infected erythrocytes in response to PPMP treatment. By intersecting these genes with those predicted to be exported to the erythrocyte and conserved across the genus *Plasmodium* [10],[15], we identify a protein that is apparently necessary for TVN assembly and stimulates endovesiculation from the erythrocyte membrane. These data suggest that although transcription by the malaria parasite is thought to be largely ‘hard-wired’, a block in erythrocyte remodeling induces a measurable transcriptional response and reveals parasite proteins that function in novel pathogenic mechanisms of nutrient acquisition in the host cell.

## Results

**Figure 1:**
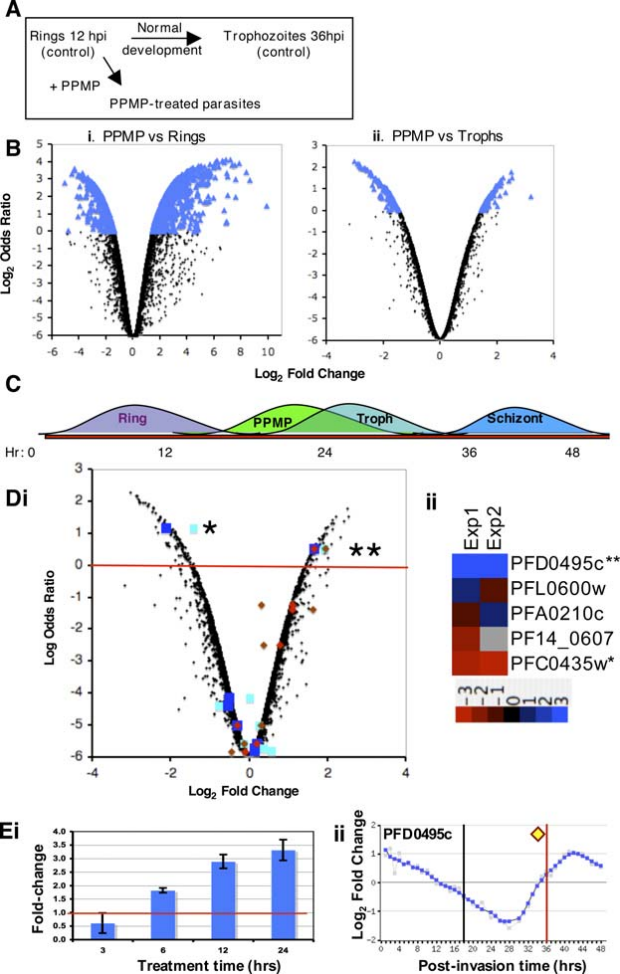
Identification of PFD0495c as a candidate gene linked to sphingolipid synthesis. (A) Schematic of *P. falciparum* maturation from rings at 12 hours post-invasion (hpi) to trophozoites at 36 hpi. *dl-threo*-PPMP inhibits parasite sphingomyelin synthase in the TVN and blocks development of TVN-tubules. For the microarray experiments both the rings and trophozoites served as controls for comparison to the PPMP-treated parasites. (B) Log odds ratio plots [32],[33] of changes in PPMP-treated parasites relative to (i) rings (starting population) or (ii) trophozoites (obtained by mock treatment). Transcriptional response of treated set is closer to the profile of trophozoite stage parasites, thus PPMP-parasites are trophozoite-like. Blue indicates genes with log odds ratios greater than zero (373 genes for rings, and 81 for trophozoites). Since the number of genes that change is greater for rings than trophozoites, the PPMP-treated parasites are more similar to trophozoites and quite distinct from rings. (C) Summary of transcriptional response suggesting PPMP-parasites are trophozoite-like. (D) (i) Log odds ratio plot of transcriptional changes in PPMP-treated parasites relative to control trophozoites. Squares indicate genes with a host-targeting (HT) motif that are conserved and syntenic across species as predicted by Hiller [8]; diamonds indicate genes with a PEXEL motif that are conserved across species as predicted by Sargeant [10], using an independent algorithm distinct from [8]. Blue squares and red diamonds represent data from experiment 1, cyan squares and brown diamonds from a biological replicate, experiment 2. Red line delineates genes with log odds ratios greater than zero. **PFD0495c is reproducibly shown to be upregulated ∼3 fold in both experiments 1 and 2 and is recognized by both Hiller and Sargeant to be exported and conserved across species. (ii) Dendrogram of microarray data sets from two biological replicates showing that of the five conserved genes of the Hiller secretome validated to be exported [15], only two are regulated by PPMP: 3-fold increase in PFD0495c(**), 2-fold decrease in PFC0435w(*). (E) qRT-PCR confirms that *pfd0495c* transcript is up-regulated by PPMP treatment. (i) Ring-stage parasites (12 hpi) were treated with 5 µM PPMP for times indicated. By 12 and 24 hr of treatment (24 and 36 hpi, respectively) *pfd0495c* transcript is significantly up-regulated compared to vehicle control. (ii) Transcriptional profile over 48 hr life cycle provided by PlasmoDB. Peak of transcription occurs at 42 hpi. Yellow diamond indicates upregulation in PPMP-treated cells after 24 h compared to corresponding normal parasites at 36h. The black and red lines indicate transcription levels of the controls used for the array: ring starting population and mock-treated trophozoite, respectively. Since PPMP-treated parasites are slightly immature compared to mock treated control, up-regulation of PFD0495c cannot be a result of growth retardation of the treated parasites.

To identify proteins linked to the TVN, we examined transcriptional changes induced in *P. falciparum* genes in response to treatment of infected erythrocytes with PPMP for 24 h. We compared PPMP-treated parasites to rings (the starting population) and trophozoites (the mock control) (Figure 1A). In the PPMP-ring comparison, 373 gene transcripts changed (Figure 1Bi), and in the PPMP-trophozoite comparison 81 transcripts changed (Figure 1Bii). Since the number of transcripts and the magnitude of change is greater in the PPMP-ring dataset relative to the PPMP-trophozoite dataset, the treated parasites have a transcriptional profile more similar to trophozoites and can be considered “trophozoite-like” (as summarized in Figure 1B and C). This is consistent with our prior data that show PPMP-treated parasites export parasite proteins and form electron dense knobs at the infected erythrocyte membrane [4], properties that are characteristic of trophozoites. Because PPMP-treated parasites are ‘trophozoite-like’, we focused on changes in transcriptional profiles relative to the trophozoite stage (Figure 1Bii).

Of the 81 genes that changed with PPMP treatment, 39 were up-regulated and 42 down-regulated. We then limited the list by focusing on gene products that met two criteria: those that contained a host-targeting signal and were conserved between human and rodent malaria parasites (Figure S1, [8],[15]). We reasoned that these proteins were likely to reflect essential functions of parasite remodeling preserved throughout the genus. An intersection of these genes with those that show PPMP-induced changes in transcriptional profiles relative to the trophozoite stage yielded two conserved genes (PFD0495c and PFC0435w; Figure 1D) of which one (PFD0495c) was up-regulated (double asterisk Figure 1Di). A biological replicate of the array experiment confirmed that these two gene transcripts were consistently modulated by PPMP treatment (Figure 1Dii). Nine other genes from the Hiller secretome and eight others from the Sergeant exportome were unchanged. In van Ooij et al. [15], we show that PFC0435w is a TVN junction protein, suggesting that our microarray data may be predictive of TVN function. To further investigate this we examined PFD0495c. It is one of 31 upregulated genes in PPMP-treated parasites whose change in expression cannot be due to developmental delay because then the transcript level would be lower than the mock-treated control (see Figure 1Eii). It is important to note that PFD0495c was recognized as a conserved protein (Figure S2) by two, independent secretome predictions, and was confirmed by RT-PCR to be specifically up-regulated by PPMP (Figure 1E). To the best of our knowledge this is the first study that has used a microarray readout from perturbation of malaria parasites to identify a candidate gene and follow up on its validation.

**Figure 2:**
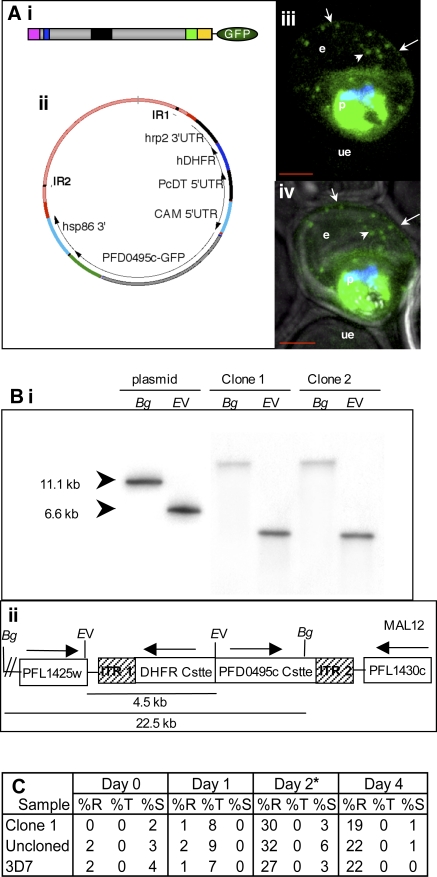
PFD0495c is exported to the erythrocyte. (A) Export of *pfd0495c-gfp* expressed as a transgene inserted into the *P. falciparum* chromosome using *piggyBac.* (Ai) C-terminal position of GFP tag and schematic of PFD0495c (ER type signal sequence (pink); host targeting motif (blue); variant repeat sequence (black); predicted transmembrane domain (green); C-terminus (yellow)). (Aii) *piggyBac* plasmid used for transfection. (Aiii) fluorescence image of *pfd0495c-gfp* expressing parasites (Aiv) fluorescence and DIC image showing export of PDF0495c chimera to the erythrocyte (e) periphery (arrow) and intraerythrocytic spots (arrowhead). (B) Site of insertion of *pfd0495c-gfp* into *P. falciparum* chromosome 12. (Bi) Southern analysis of clone 1 shows a single insertion in the genome with no evidence of episomes. Genomic DNA (2 µg) from two independent clones (1 and 2) and control plasmid DNA were digested with either BglII or EcoRV and probed with *hdhfr* coding sequence. (Bii) Insertion of the expression cassette within the *piggyBac* Inverted Terminal Repeats occurred in chromosome 12 between loci PFL1425w (T complex protein) and PFL1430c (hypothetical protein) as determined by PCR. It is remains unknown whether this is the preferred insertion site. We do not expect this site of insertion to influence export. PFL1425w is expressed in the asexual stages but does not have a predicted ER-type signal sequence so would remain within the parasite cytosol. PFL1430c is expressed in the sexual stages and to a lesser extent in asexual stages. It does have a predicted ER-type signal sequence but no host-targeting motif, so it would not be exported beyond the PVM. (C) Growth of clone 1, uncloned population, and 3D7 parasites over 4 days. Percoll purified schizonts were seeded at 2–4% schizonts in 2% hematocrit. Duplicate cultures were monitored by Giemsa-stained smears for two cycles of growth. *At Day 2, all lines were sub-cultured to 3% late rings, which matured to 3% trophs/schizonts on Day 3 and indicated parasitemia on Day 4. Counts are from duplicate experiments. Error is 10%.

To establish export of PFD0495c to the erythrocyte we utilized *piggyBac* (a type II transposon element from the lepidopteran *Trichoplusia ni*, that specifically excises and integrates at TTAA target sites) to randomly insert a tagged copy of *pfd0495c-gfp* in the genome [15],[16] (also shown for reference in Figure 2A). Integrants detected after 11 days of drug selection were cloned. Clone 1, which was selected for further characterization, showed a single site of insertion between PFL1425w and PFL1430c (Figure 2B). This insertion site is not expected to influence export. Further clone 1 showed no significant defect on *in vitro* parasite growth compared to either the uncloned population or parent 3D7 parasites (Figure 2C, Figure S3, and Protocol S1).

**Figure 3:**
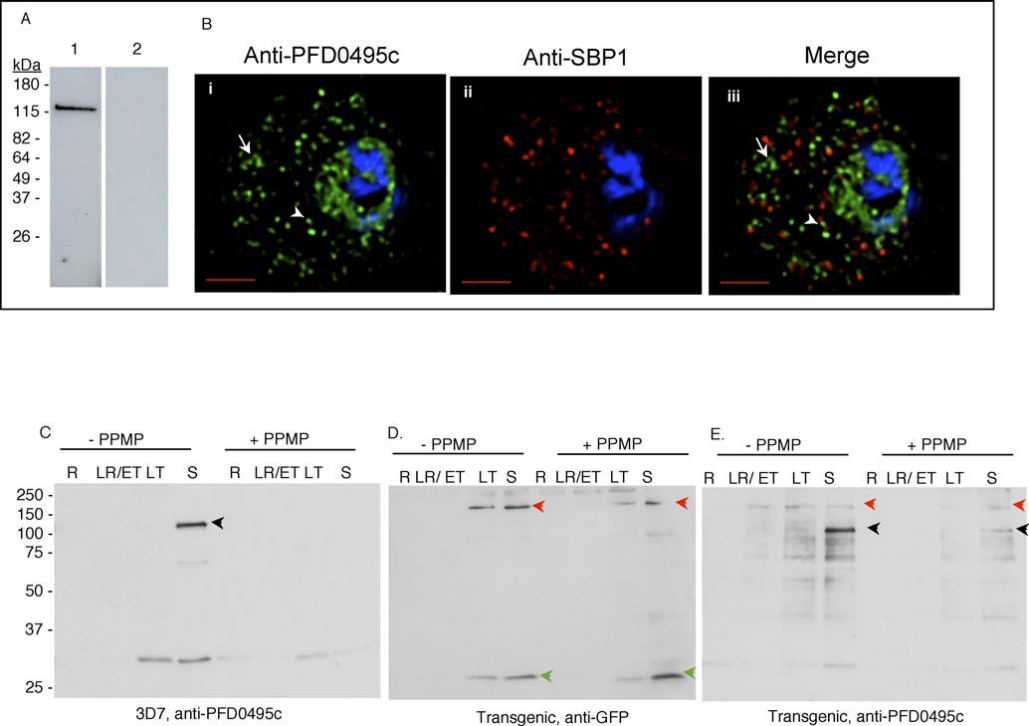
PFD0495c is found within intraerythrocytic structures, and protein synthesis of the endogenous and transgene product is in-phase and overlapping. (A) Western blots show that antibodies to the C-terminus of PFD0495c recognize a single ∼130 kDa band in infected (lane 1) but not uninfected (lane 2) erythrocytes. (B) 3D7 early schizonts were fixed and probed with antibodies to PFD0495c (i) and Skeleton Binding Protein (SBP1; ii) for immunofluorescence. Endogenous PFD0495c is found associated with the parasite and within intraerythrocytic structures, vesicular (arrowhead) and tubular (arrow) structures, distinct from Maurer's Clefts (iii). (C–E) Expression of PFD0495c in 3D7 and transgenic parasites, in presence and absence of 5 µM PPMP. Parasite equivalents of 1×10^7^ either untreated or treated with PPMP were analyzed by SDS-PAGE. 3D7 parasites were probed with antibodies to N-terminal PFD0495c (C), and the transgenic line was probed with antibodies to GFP (D) and PFD0495c (E). Antibodies to N-terminal PFD0495c recognize the endogenous protein at ∼130 kDa (of expected size) in 3D7 and transgenic parasites at schizont stages (black arrowhead). They also recognize a fusion product at ∼180 kDa in transgenic trophozoites (red arrowhead). Antibodies to GFP recognize two major bands, one at ∼180 kDa (transgene product, red arrowhead) and the other at ∼25 kDa (GFP, green arrowhead). The N-terminal PFD0495c antibody, a high titer antibody used at dilutions of 1:5000, readily detects two forms of PFD0495c in the transgenic lines when comparing all blots. The 130 kDa protein and its GFP fusion show low levels of proteolytic processing, the significance of which has yet to be determined.

Examination of *pfd0495c-gfp* parasites by fluorescence microscopy revealed fluorescence at the erythrocyte membrane (Figure 2Aiii and Aiv, arrow) and in a few intraerythrocytic structures (arrowhead) indicating that the protein was exported. However a high level of fluorescence was also detected in the parasite. To investigate whether this was a consequence of transgene expression, we used antibodies to the C-terminus of PFD0495c to examine the distribution of the native protein. As shown in Figure 3A, the antibodies recognized a single band of ∼130 kDa in infected (the predicted size of the protein is ∼100 kDa, but its apparent decrease in mobility could be due to the fact that it is transmembrane protein) but not uninfected erythrocytes. Indirect immunofluorescence microscopy revealed a high level of fluorescence associated with largely vesicular elements (Figure 3Bi, small arrowhead) localized in the intraerythrocytic space. A few tubular elements were also detected in the periphery of the erythrocyte (Figure 3Bi, arrow). A significant amount of the green fluorescence exported to the erythrocyte was distinct from the Maurer's clefts, although some was located adjacent to clefts (Figure 3Bii and Biii). Finally a significant amount of fluorescence was detected within the parasite. The precise significance of high levels of protein localization in both the parasite as well as the erythrocyte is unknown. However, the major consequence of transgene expression appears to be displacement of the tagged protein from the intraerythrocytic vesicles to the erythrocyte membrane, suggesting it may influence the dynamics of protein distribution between intraerythrocytic compartments and the erythrocyte membrane.

Transcription of *pfd0495c-gfp* is driven by the *cam* promoter, which is largely constitutive with peak transcription at the trophozoite and schizont stages [17],[18]. In contrast endogenous *pfd0495c* shows peak transcription at the schizont and early ring stages, although transcriptional activity remains detectable in late rings and trophozoites [17],[18] albeit at lower levels. We examined protein levels of PFD0495c in both 3D7 wildtype and transgenic parasites to determine how these lines differed in protein expression and their response to PPMP treatment. Although antibodies developed to the C-terminus of PFD0495c were clearly specific, they were not of sufficient titer to detect varying levels of protein throughout the asexual life cycle (not shown). We therefore developed high titer antibodies to the N-terminal repeat region of PFD0495c which also recognized one major protein band of the expected size of ∼130 kDa in 3D7 late stage schizonts (Figure 3C). Notably, this band is not detected in ring stage parasites. In the transgenic line antibodies to GFP and PFD0495c detect a protein of ∼180 kDa, which presumably corresponds to the fusion protein, in both late trophozoites and schizonts (Figure 3D and E). This is consistent with the major timing of transcript production driven by the *cam* promoter [17],[18]. Endogenous PFD0495c is also detected in the transgenic line at the schizont stage (Figure 3E). These data suggest expression of endogenous PFD0495c remains the same in transgenic and wild type parasites. The former express significant levels of fusion protein earlier (in trophozoite forms) than the endogenous protein. However, absolute levels of the fusion protein detected are small compared to amounts of endogenous protein. Further PPMP treatment appears to decrease protein expression in wild type and transgenic parasites. Hence, the modest three-fold increase in transcription from PPMP treatment (Figure 1D) does not result in a detectable increase in protein. One explanation is that *pfd0495c* is also post-transcriptionally regulated.

The lack of a detectable protein product for endogenous *pfd0495c* in ring stages (Figure 3C) even while these stages are known to be transcriptionally active (Figure S4A, B) was surprising. It is possible that either the RNA is not efficiently translated or there is high turnover of protein resulting in low levels of endogenous PFD0495c in both wild type and transgenic parasites at ring stages (Figure S4C). Hence the transcriptional/translational profile of PFD0495c is clearly distinct from a gene like RESA that is transcribed and synthesized in schizonts and carried over by daughter merozoites into the newly formed ring. The PFD0495c transcriptional profile is also distinct from MSP1, another schizont stage merozoite protein internalized upon invasion. Rather it closely mimics *pfhrpii*, a bonafide ring stage transcript, suggesting that PFD0495c is indeed transcribed (but not efficiently translated) in rings. How this transcription is linked to PPMP responsiveness is not understood. One explanation is that there are low levels of endogenous protein in 3D7 wild type parasites at almost all stages (visible upon longer exposures of the blots in Figure S4C).

**Figure 4:**
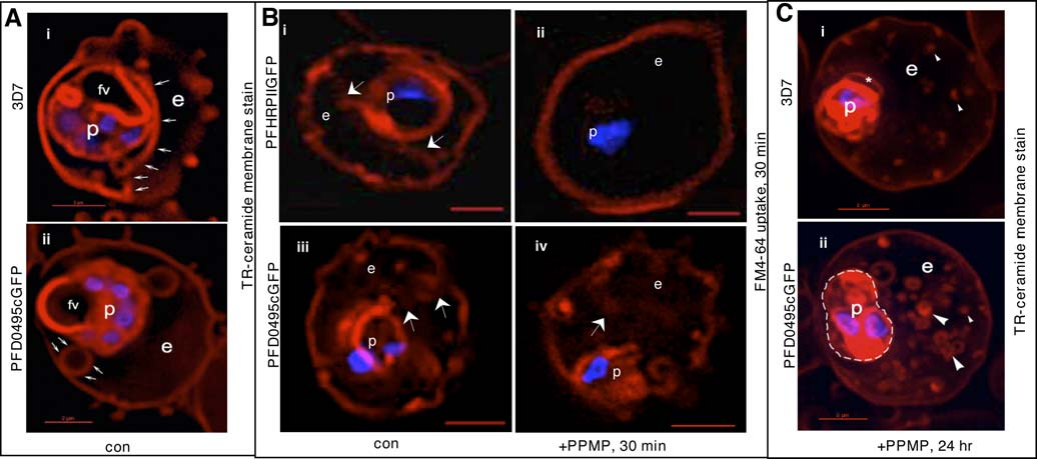
PFD0495c regulates the TVN in the infected erythrocyte cytoplasm. (A) Early schizonts (3D7 i and clone 1 parasites expressing PFD0495c-GFP ii) were stained with TR-ceramide (red; to visualize the TVN) and Hoechst 33342 (blue; to visualize DNA) and imaged live. Transgenic parasites display large TVN loops (arrows, panel ii) rather than tubules prominent in 3D7 (arrows, panel i). (B) Transgenic *P. falciparum* parasites expressing *pfhrpii-gfp* (i–ii) or *pfd0495c-gfp* (iii–iv) treated in absence (i, iii) or presence (ii, iv) of 5 µM PPMP for 30 min were incubated with membrane impermeable endocytic lipid marker FM4-64 (red) also for 30 min and imaged live. A second copy of *pfd0495c* promotes internalization of FM4-64 probe in the presence of PPMP (iv versus ii). (C) Ring stage parasites (3D7 i, *pfd0495c-gfp* clone 1 ii) were incubated with 5 µM PPMP for 24 hr, stained with TR-ceramide (red) and Hoechst (blue) and imaged live. Clone 1 accumulates an abundance of small loops and tubovesicular structures compared to few punctate spots in 3D7. Clone 1 parasites also show two nuclei (blue) and an almost two fold increase in size compared to 3D7 (that contain only one nucleus). Arrows show tubules, loops; arrowheads, vesicular structures; small arrowheads, punctate spots. For all panels erythrocyte (e), parasite (p) and intraerythrocytic structures (arrow). Scale bar, 2 µm.

Western blots in Figure 3 revealed that transgenic parasites express the fusion protein a little earlier (by the trophozoite stage) in the life cycle. Further transgenic but not wild type parasites treated with 5 µM PPMP for 24 h, display two *pfd0495c* products (transgene and endogenous) in schizont stages. Since PFD0495c associates with vesicular structures within the erythrocyte cytoplasm and its transcript is upregulated when TVN development is blocked by PPMP, we examined the TVN of transgenic parasites expressing *pfd0495c-gfp* and compared it to 3D7. We found that while 3D7 displayed characteristic tubules (Figure 4Ai small arrows), *pfd0495c-gfp* expressing parasites contained large membrane loops (Figure 4Aii), suggesting the transgene may influence TVN organization and/or function. The TVN imports lipids and raft proteins from the infected erythrocyte surface, a function that can be can be blocked by preventing TVN-tubule development with PPMP treatment [3]. As shown (Figure 4Bi) infected erythrocytes expressing the non-specific transgene *pfhrpii-gfp* (or 3D7 parasites; not shown) internalize a membrane-impermeable endocytic lipid marker FM4-64 [19] via TVN tubules, and this import can be blocked by short-term (30 min) treatment with PPMP (Figure 4Bii). However, clone 1 expressing a second copy of *pfd0495c* continues to internalize FM4-64 despite treatment with PPMP (Figure 4Biv *vs*. Bii). After long term (24h) treatment with PPMP labeling of *all* intraerythrocytic membranes with a membrane-permeable lipid, BODIPY-TR-ceramide, revealed elevated accumulation of numerous membrane loops in the erythrocyte cytoplasm in clone 1-infected cells, compared to residual intraerythrocytic structures seen in their 3D7 counterparts (Figure 4Ci versus Cii and see Figure S5). This difference in TVN organization was not due to changes in parasite sphingomyelin synthase activity inhibited by PPMP (Figure S3) or transfection per se (Figure 4Bi–ii; [20]). In addition, the effects of PPMP on parasite growth in the parent line and multiple transgenic clones were completely reversible upon washing out drug (Figure S3). This suggests that membrane accumulation seen in Figure 4Cii is not due to non-specific degeneration of intraerythrocytic structures as a consequence of parasite death but due to the presence of a second copy of *pfd0495c* in the genome. Together these data support the idea that PFD0495c changes lipid transport at the infected erythrocyte membrane. This lipid can be delivered to the parasite independent of sphingomyelin synthase activity and modulates tubular assembly in the network (Figure 4Biv, 4Cii). However, transgenic parasites appear to be less sensitive to the effects of PPMP compared to 3D7. Although they are decreased in size compared to untreated counterparts, they are slightly larger than treated 3D7 and consistently show two nuclei rather than just one nucleus seen in treated 3D7 (Figure 4Cii versus 4Ci). This may be explained by the fact that transgenic parasites express higher levels of PFD0495c protein in presence of PPMP.

**Figure 5:**
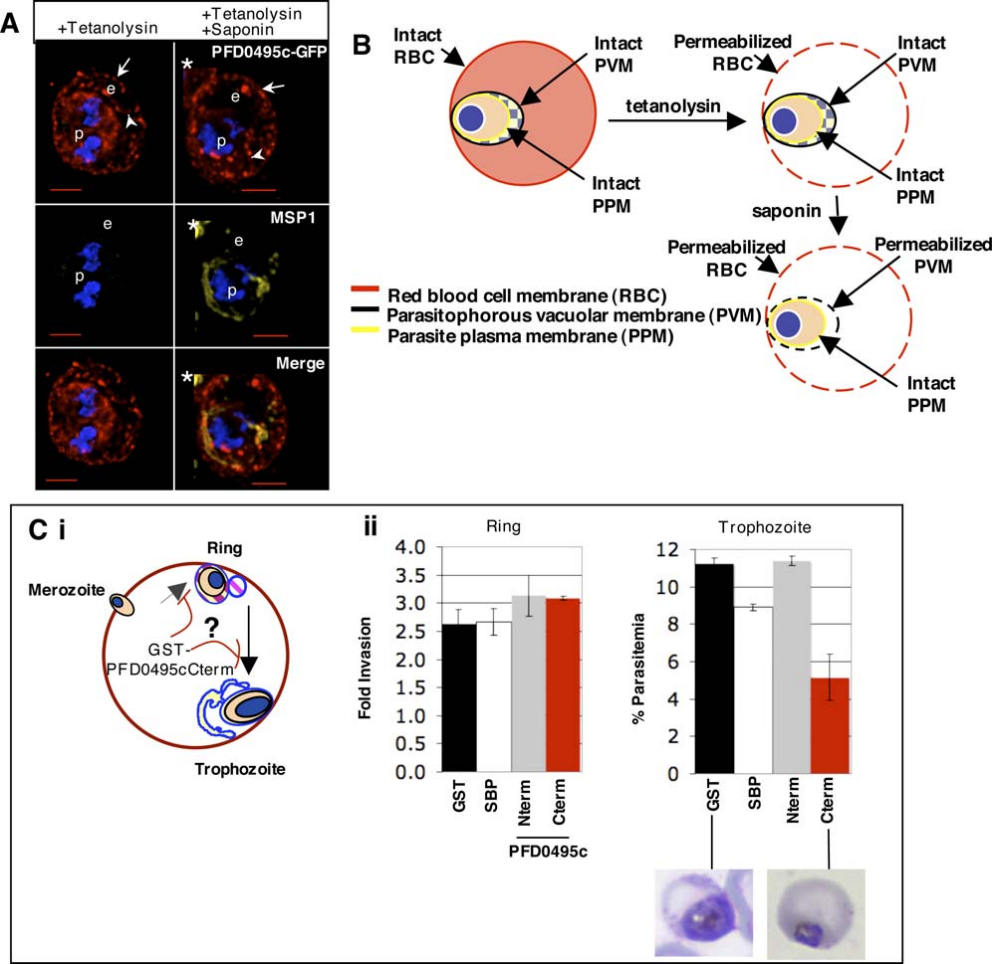
The C-terminus of PFD0495c functions within the erythrocyte cytoplasm and mediates interactions important for intraerythrocytic development. (A) Topology of PFD0495c in transgenic clone 1 parasites. C-terminus of PFD0495c-GFP is localized to erythrocyte cytoplasmic face. Anti-GFP signal is detected after tetanolysin permeabilization. Control signal to PPM (αMSP-1) is detected only with saponin treatment. p, parasite; e, erythrocyte; arrow, erythrocyte membrane; arrowhead, intraerythrocytic vesicle/tubule. Asterisk marks neighboring cell. (B) Schematic of selective permeabilization experiment. Tetanolysin selectively permeabilizes only the erythrocyte plasma membrane, leaving the PVM intact. Only in combination with saponin will the PVM be permeabilized and control MSP1 antibodies gain access to epitope. If anti-GFP signal can be detected with tetanolysin alone, then the C-terminus is present on the cytoplasmic face of the erythrocyte. (Ci) Strategy designed to target PFD0495c cytoplasmic interactions in 3D7 infected erythrocyte ghosts to identify whether this blocks either parasite invasion or intraerythrocytic growth. GST-fusions of PFD0495c C-terminus loaded into re-sealed erythrocyte ghosts block interactions of the endogenous protein on the cytoplasmic face of the developing PVM-TVN. The strategy does not require any prior information about the function of a protein and thus provides a powerful tool to annotate functions of ‘hypothetical’ genes in the *P. falciparum* genome. (Cii) Resealed erythrocyte ghosts were loaded with 50 µM GST-PFD0495cCterm (Cterm), GST-PFD0495cNterm (Nterm), GST-SBPCterm (SBP) or GST alone (GST), infected with 3D7 *P. falciparum* at 2% schizonts and ring (R), trophozoite (T), schizonts (S) were monitored after 24 h (Ring), 48h (Trophozoite). Giemsa-stained smears indicate parasite morphology shown below.

Next we were interested in determining whether PFD0495c is essential for TVN development. It is one of only 11 conserved, exported proteins encoded by syntenic genes in *Plasmodium* genomes. We attempted to delete nine of the 11 genes in *P. berghei* but were unsuccessful [15], suggesting that they may be essential genes. Thus independent evidence that PFD0495c contributed to the TVN formation/function would likely not be obtained by undertaking knock out experiments. We therefore chose an alternate approach of loading cargo into resealed erythrocytes to monitor for effects on parasite invasion or growth. The principle is that by loading dominant-negative recombinant fragments of the protein of interest into ghosted erythrocytes and infecting, endogenous protein interactions will be disrupted, which will have effects on parasite fitness. Only proteins that function within the erythrocyte cytosol may be considered, and the domains that function within the erythrocyte cytosol must be identified. This approach has been employed to identify known cytoplasmic determinants of both host and parasite origin needed for parasite invasion [7],[21] and has recently been optimized to support parasite invasion and growth to the same degree as normal erythrocytes [21]. To extend it to investigation of a protein of unknown function, we needed to identify which domain of the protein was exposed to the erythrocyte cytoplasm. As shown in Figure 5, selective permeabilization of the erythrocyte membrane by tetanolysin, which leaves vacuolar and intraerythrocytic structures intact, suggested that the C-terminus of PFD0495c is cytoplasmically oriented in the erythrocyte of a late trophozoite/schizont-infected cell (Figure 5A and B).

To assess the functional importance of this domain during infection, we introduced 50 µM of a recombinant form of the C-terminal region of PFD0495c fused to glutathione S-transferase (GST-PFD0495cCterm; Figure S6) into the cytoplasm of resealed erythrocyte ghosts [21] (Figure 5Ci). Introduction of GST-PFD0495cCterm had no significant effect on invasion (measured as ring formation), but was clearly inhibitory to trophozoite growth. GST alone or a recombinant fusion of a parasite protein domain (PfSBPCterm) known to be exposed to the erythrocyte cytoplasm but not required for infection [12] had no effect (Figure 5 Cii). In addition, an N-terminal fragment (GST-Nterm) that is thought to be lumenal has no effect on parasite growth (Figure 5 Cii), which further validates the topology. High micromolar concentrations of recombinant fragments used in these experiments have been used in other studies at concentrations of peptides and protein domains (40–80 µM; [7],[21]) that inhibit functions of known host and parasite proteins in resealed ghosts. Further the cytoplasmic C-terminus of both SBP and PFD0495c are highly polar, a feature which is also shared by predicted cytoplasmic regions of many intraerythrocytic parasite proteins [22]. Thus inhibition of parasite growth seen by GST-PFD0495cCterm appears to be a sequence-specific effect, likely due to its interference with the actions of the endogenous protein and not due to non-specific effects of charge.

**Figure 6:**
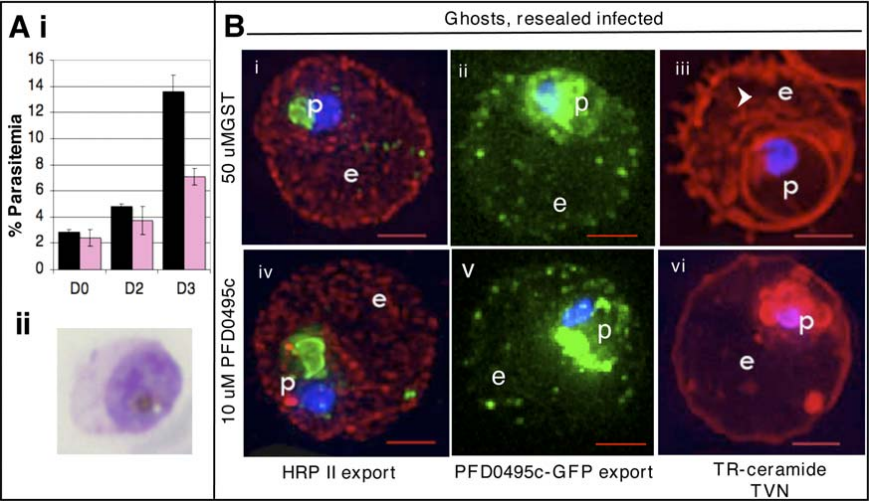
PFD0495c is important for proper development of the TVN. (Ai) Erythrocyte ghosts were resealed with 10 µM GST-PFD0495cCterm (pink bars) or 50 µM GST alone (black bars) and infected with *P. falciparum* to achieve ring parasitemias of 2–3% on Day 0 (D0). On Day 2 in ghosts loaded with 50 µM GST, trophozoites, schizonts and a few new rings were observed. However in D2 ghosts loaded with 10 µM GST-PFD0495cCterm, only trophozoites were seen (see Aii). On Day 3 (D3), parasitemia in ghosts loaded with GST alone was 12–14% rings, while in ghosts with 10 µM GST-PFD0495cCterm it went up to only 6–7%. (B) Trophozoite stage parasites in ghosts resealed with 50 µM GST (Bi, ii, iii), or 10 µM GST-PFD0495cCterm (Biv, v, vi), were infected with: (Bi, iv) 3D7 *P. falciparum* and probed with anti-HRPII (red), anti-MSP1 (green, to mark the parasite) and Hoechst 33342 (blue to stain parasite DNA); (Bii, v) clone 1 expressing PFD0495c-GFP and visualized live for green fluorescence; (Biii, vi) 3D7 *P. falciparum* stained with the membrane permeable probe TR-ceramide and visualized live.

**Figure 7:**
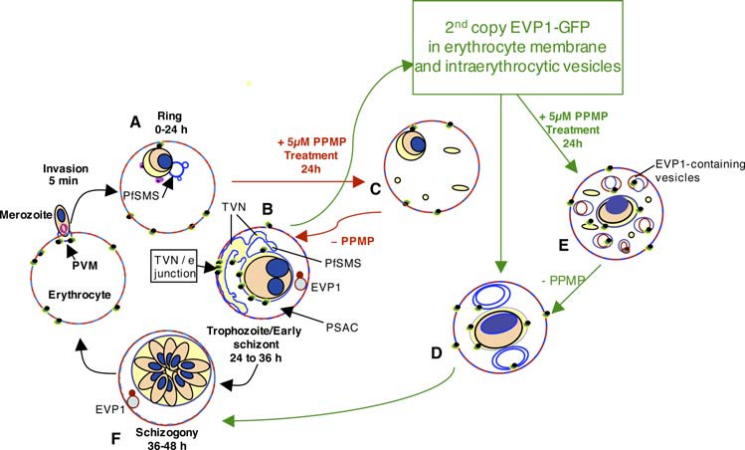
Proposed model for involvement of erythrocyte and parasite rafts, sphingolipid synthesis in the PVM-TVN and EVP1. Rings (A) bud nascent TVN vesicles (blue) that, in the presence of sphingomyelin synthesis, stabilize into tubules (blue) at the trophozoite stage (B). *dl-threo*-PPMP inhibits sphingomyelin synthesis in the TVN and blocks development of TVN-tubules (C). Expression of EVP1-GFP stimulates large loops (not tubules) in TVN of trophozoites and schizonts (D). Treatment of transgenic cells with *dl-threo*-PPMP induces many small loops and vesicles in the erythrocyte (E). These vesicles are stained by a membrane impermeable dye applied to the surface of infected erythrocytes that is usually excluded from uptake in PPMP-treated cells. EVP1 localizes to these vesicles within the erythrocyte shown in red in panels B, E and F. We suggest that EVP1 drives vesicular lipid uptake at the infected erythrocyte membrane, and sphingomyelin synthase drives tubule formation and lipid uptake via tubules possibly to increase the efficiency of uptake. PSAC, Parasite surface anion channel (PSAC) for solute import [5]. TVN/erythrocyte (TVN/e) junction used by host raft proteins and lipids use to directly access the TVN.

To investigate whether inhibition of trophozoite maturation could be specifically linked to intraerythrocytic transport functions, we reduced the concentration of GST-PFD0495cCterm in the erythrocyte cytoplasm to 10 µM. This allowed growth of enlarged trophozoites (see Figure 6 Ai–ii) and had no deleterious effect on parasite protein export (Figure 6Bi and iv; ii and v). However, GST-PFD0495cCterm clearly disrupted tubular-TVN development (compare Figure 6Biii and vi) as well as the next cycle of parasite growth (Figure 6A), consistent with a defect in maturation of trophozoites to the schizont stage (not shown). Together these data suggest that introduction of PFD0495c C-terminus disrupted TVN assembly. This was not a consequence of non-specifically blocking parasite protein export to the erythrocyte. Rather it is likely to specifically block cytoplasmic interactions of PFD0495c, which mediates important interactions required for stable maintenance of the TVN. These data reveal that PFD0495c marks a unique vesicular compartment in the infected erythrocyte cytoplasm and that its function is linked to the TVN (Figure 7, where PFD0495c is delineated as Erythrocyte Vesicle Protein 1).

## Discussion

The first parasite determinant (a sphingolipid synthase activity) known to be important to the TVN was identified in the mid-nineties concomitant with the identification of this organelle [23]. Despite steady progress on characterizing the function of the TVN in the import of nutrients, lipids and host raft proteins [3],[4],[24], the contribution of additional parasite proteins to the network has remained elusive. Almost fifteen years after the discovery of the synthase we identified two candidate genes PFC0435w and PFD0495c that may be linked to the TVN. PFC0435w is shown to be a TVN junction protein [15] while PFD0495c defines a novel vesicular membrane compartment in the infected host cell (and we therefore name it erythrocyte vesicle protein 1 or EVP1) that shifts lipid dynamics within the host cell. The discoveries of EVP1 as well as TVN Junction Protein 1 (TVN-JP1; [15]) well over a decade after the identification of the sphingolipid synthase was achieved by integrating expression profiling with additional rapid genetic and functional assays.

The finding that microarray outputs of malaria parasites may be predictive for function is somewhat contrary to the idea that has emerged from prior work that the parasite is hard-wired and does not respond to environmental cues [25]. One reason for the discrepancy may be that most studies investigate transcriptional change in response to drugs that kill the parasite. The secondary effects of death may confound analysis by generating noise that is difficult to filter out. In contrast PPMP treatments for periods used in these studies (24 h) arrest parasites at the early trophozoite stage but do not kill them. Additionally, because secretome gene products are at the host-pathogen interface and function between the intracellular parasite and the blood, they may be more sensitive to environmental cues. The fact that PFD0495c protein levels did not increase concomitantly with PPMP up-regulation of transcription may be due to several reasons. One explanation for this discrepancy is that the increase in transcript levels is too modest to be able to detect an increase in protein. A second possibility is that the high molecular weight oligomeric forms inhibit detection of protein at the correct molecular weight. A third alternative is that *pfd0495c* messenger RNA is degraded faster than protein can be made. In fact, transcription of EVP-1 is detected throughout the lifecycle, dipping during the late trophozoite stage and peaking at late schizogony (Figure 1Eii; [17],[18]). Shock and colleagues recently undertook global analysis of steady-state mRNA levels and determined that the half-life of a transcript is shortest in ring stages and longest in schizont stages [26]. The half-life of EVP-1 is 7 min in rings and 92 min in schizonts [26]. Perhaps a longer half-life is required for efficient translation of *evp-1* message. Lastly, higher protein turn-over could offer another explanation. Since Westerns measure steady-state levels, it is possible that more protein is synthesized but also rapidly degraded.

Notably, although *pfd0495c* is up-regulated with PPMP treatment, schizont stage genes, such as MSP-1, are not. This suggests that our treated parasite population is well synchronized and not contaminated with parasites that are beyond the PPMP block. In fact, MSP-1 is down-regulated with PPMP treatment indicating that the parasites do not progress through schizogony. Although PPMP-induced changes are relatively modest, they were predictive for TVN function of our candidate genes. The use of microarrays as predictive tools is enormously beneficial in human malaria parasites that are difficult to manipulate genetically.

Our studies provide a road map to rapidly move from *in silico* predictions to initial annotation of these proteins in infection (Figure S7). *piggyBac* provides a rapid system to produce transgenic parasites with which to examine changes in intraerythrocytic transport functions in antigen export and lipid/nutrient import. Producing these parasites with such speed enables detailed characterization of sites of integration as well as other characteristics to ensure that they are suitable for analysis of a given transport phenotype. Finally for genes likely to be essential, our study provides an alternative approach to assessing functional importance of their gene products for invasion or intracellular parasite growth. This method relies on the ability to disrupt endogenous protein interactions by loading dominant-negative recombinant forms into resealed erythrocyte ghosts. The use of small peptide domains (of 30–90 amino acids) in GST fusions circumvents difficulties in expressing recombinant forms of *P. falciparum* proteins without codon optimization and is consistent with published data that small protein domains of *P. falciparum* are efficiently expressed as soluble protein in *E. coli* [27]. Larger, more insoluble domains can be truncated into smaller domains to identify specific inhibitory portions. Truncations in conjunction with secondary structure prediction and production of overlapping fragments can assist in production of optimal fragments (Murphy and Haldar, unpublished). In principle this strategy may be applicable to over 50% of parasite genes involved in pathogen-host interactions where over half have unknown functions [28].

Our studies of EVP1 suggest that its associated vesicles are distinct from Maurer's clefts known to function in protein export to the erythrocyte. The distribution of EVP1 is also distinct from PVM proteins such as PfEXP1 and ETRAMPs that are seen in membrane extensions off the PVM. In this context the intraerythrocytic compartment to which EVP1 localizes may define a hitherto poorly defined membrane intermediate in the infected erythrocyte. Our initial localization studies suggest the bulk of these vesicles are not at the erythrocyte periphery, although the gene has an elevated rate of polymorphisms [29]–[31], suggesting it is under host pressure. Further expression of a second copy of the gene can change the dynamics of localization, suggesting function in a novel endovesiculation pathway or transbilayer lipid movement at the infected erythrocyte membrane. We speculate that the physiological substrate of EVP-1 could be a variety of lipids, including sphingolipids, although a definitive substrate remains to be identified. Nevertheless changing lipid import properties at the infected erythrocyte membrane does not circumvent the block induced by PPMP (although the parasites are arrested at a state of two rather than one nucleus), suggesting additional factors also contribute to proper TVN and membrane import. We can begin to piece together the sequence of events that build and maintain the TVN. Sphingomyelin synthesis is a critical early event. If its activity is inhibited early in the asexual cycle, then the TVN is not properly assembled and nutrients are not imported. Several pieces of evidence support the idea that EVP1 is required later in the cycle. The predominant form of the protein is detected in later stages and not in ring stages. The resealed ghosted erythrocyte experiments demonstrate that the dominant-negative GST recombinant form of the C-terminus inhibits parasite maturation not invasion. Finally the transgenic line retains sensitivity to PPMP but has altered TVN properties. Since the TVN is assembled from the late ring stage to early schizogony, the data unexpectedly reveal that vesicular import from the erythrocyte membrane at its later stages of assembly may be important for the maintenance of this network. Hence the TVN remains a dynamic structure even though its tubules appear as relatively immobile structures in the erythrocyte cytoplasm.

The facts that EVP1 is important for the development of the TVN and is also a member of a conserved set of exported proteins suggest that the encoding gene *pfd0495c* is essential for blood stage infection. Finally, although essential genes cannot yet be knocked out in blood stage *Plasmodium*, we nonetheless show that this does not preclude insight into the complexity of vesicular intermediates and membrane dynamics utilized by a pathogen to develop an essential nutrient transport pathway in its mammalian host.

## Materials and Methods

### Transcriptional response of *Plasmodium falciparum*-infected erythrocytes to PPMP treatment

Ring stage parasites were treated with either 5 µM *dl-threo*-PPMP or vehicle ([ethanol]_f_ = 0.01%) for 24 hr and were harvested for RNA isolation. Untreated ring stage parasites were also harvested. The experimental design served to compare PPMP- to either mock-treated or ring stage transcripts. This first biological replicate compared treated to control directly, whereas the second biological experiment compared each sample (treated or control) to a reference sample, pooled RNA. RNA was isolated according to Invitrogen (Carlsbad, CA) protocols using Trizol. First strand cDNA synthesis and hybridizations were performed according to previous protocols [18]. Microarray slides were scanned using GenePix 4000B Scanner and analyzed with GenePix Pro 4.1. The statistical program R with the add-in package SMA (from Terry Speed's group at http://www.stat.berkeley.edu/7Eterry/zarray/Software/smacode.html) was used to normalize data [32],[33] for the first biological experiment. Features with a log odds ratio above zero were considered. Only high quality features and those with signal two standard deviations above background were analyzed for both biological replicates, leaving 4580 elements from a total of 8088 oligos for the first and 6221 elements from a total of 7283 oligos for the second experiment.

### Quantitative RT-PCR

Quantitative PCR [34] was used to confirm the array results. Parasites were synchronized by successive rounds of percoll and sorbitol. Ring stage parasites were treated with either 5 µM *dl-threo*-PPMP or vehicle (ethanol = 0.01%) for 3, 6, 12, and 24 h. RNA was isolated using Trizol (Invitrogen) and treated with DNAse (Promega) according to manufacturer's recommendations. Integrity of the RNA was confirmed with the 2100 Bioanalyzer (Agilent). First strand cDNA synthesis was initiated by priming 5 µg RNA with 40 µg/mL oligo(dT)_12–18_ at 65°C for 5 min then incubating reaction with 0.5 mM dNTPs and 200 U SuperScript reverse transcriptase (Invitrogen) for 60 min at 42°C. The reaction was inactivated by heating to 70°C for 15 min. Each qRT-PCR reaction, done in triplicate, contained 1 ng cDNA, 2 µM primers (forward 5′-GCTCTTTCCATAAATACTGTATT-3′, reverse 5′-ATGGCCAAACAACATCA-3′) and SYBR green chemistry (Applied Biosystems) and was done using the 7900HT ABI system according to ABI protocols. Amplification of *P. falciparum* 18S ribosomal RNA (gi 160642), an endogenous control, was done in parallel (forward 5′-ACAATTGGAGGGCAAGT-3′, reverse 5′-TTGGAGCTGGAATTACC-3′) to standardize the amount of sample in each well. A control reaction that did not receive reverse transcriptase was included to account for non-specific amplification due to contaminating DNA. Relative quantification was performed using the comparative method, whereby the amount of *pfd0495c* was normalized to the endogenous control 18S rRNA. Control samples receiving only vehicle were used to calibrate each PPMP sample at that time point.

### Transfection using *PiggyBac* type II transposable element

Full length *pfd0495c* (gi 23510091) was amplified from gDNA using the oligos 5′-aaaaagcaggcttcgaaggagatagaaccatgATGTATAAGAAATGTTTCATTTTATATCCTATCTTTTTTC-3′ and 5′-agaaagctgggtcTCATCTGTCGTCGGAACGGAAGGAATC-3′ (partial attB sites in lower case). Cloning with the Gateway system was according to Invitrogen protocols. To make the destination vector, the dhfr gene with control regions and *pfcam* promoter and *pfhsp86* 3′ UTR was ligated to pXL-BAC-HH [16], which contained OriC, the ampicilin resistance gene and the IR and TR sequences. The transposase plasmid was described previously [16].

Both integration and transposase plasmids (100 µg each) were simultaneously electroporated into erythrocytes. Forty-eight hours after transfection, selection with 2.5 nM WR99210 was initiated. After 11 days of selection, GFP-expressing parasites were detected. GFP-expressing parasites were then cloned by limiting dilution at 0.2 parasites/well in a 96-well plate. Fresh media and 1% hematocrit were added at days 6 and 13, and at day 17 parasitemia of each well was determined by examining thick smears stained by Giemsa.

### *PiggyBac* insertion site analysis

To confirm integration into the genome, Southern analysis was performed, digesting 2 µg DNA (either gDNA or plasmid) with 10 units of either BglII or EcoRV. The coding sequence of *hdhfr* labeled with ^32^P was used as a probe. No episomes were maintained, and only one insertion was detected for both clones 1 and 2 of PFD0495c-GFP. *piggyBac* insertion sites in the genome were identified by using an adaptor-ligation-mediated PCR method (Balu and Adams, unpublished data). Briefly, Rsa I digested genomic DNA was ligated to compatible adaptors and used in a PCR reaction with an internal piggyBac primer and a primer in the adaptor. The PCR products obtained were then directly sequenced to identify the insertion sites. Insertion of the expression cassette within the *piggyBac* Inverted Terminal Repeats occurred in chromosome 12 between loci PFL1425w (T complex protein) and PFL1430c (hypothetical protein).

### Growth of PFD0495c-GFP transgenic line and 3D7

To compare growth of *pfd0495c-gfp* expressing parasites to 3D7, Giemsa-stained blood smears were counted to determine numbers of rings, trophozoites and schizonts. Percoll-purified schizonts were mixed with erythrocytes at ∼2% parasitemia in 2% hematocrit. Blood smears were made every 24 hr from day 0 to 4, and medium was changed daily. At day 2 ring stage parasites were subcultured to 3% parasitemia. The experiment was conducted in duplicate, and the counter was blinded to sample identity. There were no detectable differences in growth among the uncloned population, clone 1 PFD0495c-GFP, and 3D7 parasites.

### Antibodies to PFD0495c

In rabbits, anti-peptide antibodies were raised against the C-terminus of PFD0495c: peptide LKFQHDQEFLNYFKRYQDFN (NeoMPS, San Diego, CA). Lysates of 1×10^6^ parasite or uninfected erythrocyte equivalents were analyzed by SDS-PAGE and probed. Antibodies reacted with a single band only in parasite lysates.

Anti-peptide antibodies were raised against the N-terminal repeat region of PFD0495c: peptide DDNVNHTNDDKVNHTN (Affinity Bioreagents, Golden, CO). Infected cell pellets were lysed in hypotonic buffer (10 mM Tris, 2 mM EDTA, 2 mM EGTA, pH 7.4) and centrifuged at 100,000 × *g* to pellet membranes. Pellet fractions of 1×10^7^ parasite equivalents were separated by SDS-PAGE and probed with indicated antibodies.

### Immunofluorescence

To determine localization of endogenous PFD0495c, 3D7 trophozoites were harvested and probed with antibodies to PFD0495c and Skeleton Binding Protein (SBP1) and appropriate secondary antibodies conjugated to FITC or rhodamine. Nuclei were stained with 10 µg/mL Hoechst 33342. Images were captured using DeltaVision Deconvolution microscopy as described [3]. A high-resolution 3-D image was captured with an Olympus IX inverted fluorescence microscope and a Photometrix cooled CCD camera (CH350/LCCD) driven by DeltaVision software (softWoRx) from Applied Precision Inc (Seattle, WA). Twelve to fifteen 200 nm optical sections were taken through the depth of the cell, and DeltaVision software was used to deconvolve images and reconstruct a 3-D view.

To determine effects on export in resealed erythrocytes, ghosts that were infected with 3D7 were probed with antibodies to HRPII (Santa Cruz) and MSP1 (MR4) and appropriate secondary antibodies conjugated to FITC or rhodamine. Nuclei were stained with 10 µg/mL Hoechst 33342. Cells were viewed using DeltaVision Deconvolution microscopy as described [3].

### Monitoring endocytic lipid import with the lipid marker FM4-64

FM4-64 (*N*-(3-triethylammoniumpropyl)-4-(6-(4-(diethylamino)phenyl)hexatrienyl)pyridinium dibromide) is a fluorescent lipid marker that has been used to study endocytosis in eukaryotic cells [19]. To visualize active uptake of FM4-64 from the erythrocyte plasma membrane during intraerythrocytic growth, trophozoite stage parasites expressing either PfHRPII-GFP or PFD0495c-GFP were incubated in the absence or presence of 5 µM *dl-threo*-PPMP for 30 min at 37°C. Infected erythrocytes were washed three times in PBS then stained with 16 µM FM4-64 for 30 min at 37°C. Nuclei were stained with 10 µg/mL Hoechst 33342, and cells were washed with PBS three times. Cells were imaged live with DeltaVision Deconvolution microscopy [3].

### Visualization of tubo-vesicular network by BODIPY-Texas Red C_5_ Ceramide staining

To visualize TVN membranes, erythrocytes or ghosts infected with indicated *P. falciparum* strain were washed free of serum and stained with 2.5 µM BODIPY-Texas Red C_5_ ceramide (*N*-((4-(4,4-difluoro-5-(2-thienyl)-4-bora-3a,4a-diaza-*s*-indacene-3-yl)phenoxy)acetyl)sphingosine, Invitrogen) for 15 min at 37°C. Nuclei were stained with 10 µg/mL Hoechst 33342. The cells were washed in RPMI 1640 three times and viewed live by DeltaVision Deconvolution microscopy as described [3].

### Determination of PFD0495c-GFP topology using Tetanolysin

Selective permeabilization of the erythrocyte plasma membrane using tetanolysin was performed according to published protocols [35]. Erythrocytes infected with PFD0495c-GFP expressing parasites (38–42-h post-invasion) were probed with antibodies to GFP (Molecular Probes) or MSP1 (Anthony Holder) and appropriate secondary antibodies conjugated to rhodamine or Cy-5. Cells were viewed using DeltaVision Deconvolution microscopy as described [3].

### Expression of GST fusions

To produce recombinant GST-PFD0495c cargo, the C-terminus (amino acids 795 to 833) was amplified using oligonucleotides 5′-aaaaagcaggcttcGATTTAGATATTGATGATACTTTAAAGTTTCAGCATGATCAA-3′ and 5′-agaaagctgggtcTCATCTGTCGTCGGAACGGAAGGAATC-3′ (partial attB sites in lowercase). To produce GST-Repeat cargo, the repeat region was amplified using oligonucleotides 5′- aaaaagcaggcttcGATGATGTGGTGAGAAATATTAACGATGATGTG-3′ and 5′ agaaagctgggtcTCAACTATTATTAGTTTTTATATCACCTGCATTATTCTTTTTATCATTATA-3′. To produce GST-SBP cargo, the C-terminus of SBP (amino acids 239 to 337) was amplified using oligos 5′-aaaaagcaggcttcGGAAAAAGAAAAGGATATTACCTAGCAAAAAAAC-3′ and 5′-agaaagctgggtcTTAAGGTTTCTCTAGCAACTGTTTTTGTTGTGG-3′. Cloning into the Gateway system was performed with pDEST15 (Invitrogen). Expression of recombinant fusions was induced with 1 mM Isopropyl-(beta)-D-thiogalactopyranoside (IPTG) (Eppendorf) for 2 h at 37°C. Protein purification was performed with glutathione resin (Clontech) in non-denaturing buffer containing 50 mM Tris, pH 8, 50 mM NaCl, 5 mM EDTA.

### Loading GST-fusions into erythrocyte cytoplasm and subsequent infection by *Plasmodium falciparum*

Erythrocytes were loaded with GST cargo and resealed according to previous protocols [21]. Schizonts were percoll-purified to >95% purity and mixed with loaded ghosts at 2% parasitemia. Blood smears were made from a hematocrit of 20–30% and stained with Giemsa for 7 minutes, rather than 2 min, in order to better visualize ghosts. Numbers of rings, trophozoites and schizonts were enumerated by a counter who was blinded to sample identity. Parasite morphology was monitored for any differences. Images of blood smears were taken by light microscopy with a Zeiss Axioskop upright microscope and Nuance spectral camera/un-mixing system (Cambridge Research and Instrumentation).

## Supporting Information

Protocol S1 Supplementary Materials/Methods and References
(0.05 MB DOC)

Figure S1 List of genes containing a host-targeting motif conserved between *P. falciparum* and *P. berghei* [3].
(0.38 MB TIF)

Figure S2 Sequence of PFD0495c.
(2.13 MB TIF)

Figure S3 Comparative analyses of effects of PPMP on maturation of parent 3D7 and clones 1 and 2 expressing PFD0495c-GFP.
(2.89 MB TIF)

Figure S4 Detailed analysis of microarray and protein expression data of PFD0495c.
(4.08 MB TIF)

Figure S5 Quantification of parasite-induced structures within the erythrocyte cytosol in 3D7 wild-type and PFD0495c-GFP transgenic parasites treated with 5 µM PPMP for 24 h.
(1.33 MB TIF)

Figure S6 Coomassie-stained SDS-PAGE gels of GST-fusions of C-terminal domains of PFD0495c and SBP, and N-terminal PFD0495c repeat region expressed in *E. coli* (>90% purity) purified under native conditions.
(1.46 MB TIF)

Figure S7 Flow diagram of strategy to identify and functionally characterize *P. falciparum* proteins exported to the erythrocyte and required for blood stage parasite infection.
(0.99 MB TIF)
