# Supplementary material for: An Erythrocyte Vesicle Protein Exported by the Malaria Parasite Promotes Tubovesicular Lipid Import from the Host Cell Surface
Source: PLoS Pathog. 2008 Aug 8;4(8):e1000118. doi: 10.1371/journal.ppat.1000118 (PMC2483944; doi:10.1371/journal.ppat.1000118)
Supplement: Protocol S1 — Supplementary Materials/Methods and References (0.05 MB DOC) [file ppat.1000118.s001.doc]

**Supplemental Material for:**

An erythrocyte vesicle protein exported by the malaria parasite promotes tubovesicular lipid import from the host cell surface.

Pamela A. Tamez, Souvik Bhattacharjee, Christiaan van Ooij, N. Luisa Hiller, Manuel Llinás, Bharath Balu, John H. Adams, and Kasturi Haldar^*^

^*^To whom correspondence should be addressed. Email: k-haldar@northwestern.edu

**Materials and Methods**

PlasmoDB ([http://www.plasmodb.org](http://www.plasmodb.org/)) provided sequence information and transcriptional plots [1].

**Measurements of IC_50_ and IC_90_ of PPMP in PFD0495c-GFP transgenic line and 3D7 strains**

Our previous studies have shown that PPMP is a sphingolipid analog that inhibits an essential parasite sphingomyelin synthase (PfSMS) activity exported to the erythrocyte and after 36 h of treatment, the effects of this inhibitor are cidal with low micromolar concentrations inhibiting 50% and 90% parasite growth [2]. A change in activity levels of exported PfSMS activity is thus expected to be reflected in the IC50 and/or IC90. Hence the concentrations to inhibit 50% and 90% growth in parent and transgenic lines were determined by standard hypoxanthine incorporation assay [2]. Synchronized ring stage parasites were treated with PPMP over a concentration range of 0.02-20 uM at 0.6% pst and 1% hct in hypoxanthine-free CRPMI. After incubating for 24 hr, 0.5 uCi [8-^3^H]Hypoxanthine (GE Biosciences) was added to each well. After an18 hr incubation infected erythrocytes were harvested onto glass fiber filters, which were dried and counted. All data were regressed using the logistic dose response function of Tablecurve 2D software (Systat, [http://www.systat.com](http://www.systat.com/)).

**Reversibility of treatment with PPMP after 24 hr**

Synchronized ring stage parasites at 5-10% pst were incubated with 5 uM PPMP for 24 hr (day 1). Infected erythrocytes were washed with RPMI 1640 three times to remove PPMP then put back into culture. Blood smears were made at days 0, 1, 2, and 3 and stained with Giemsa. Parasites were fed fresh media every 24 hr. Rings, trophozoites and schizonts were enumerated by a counter who was blinded to sample identity. Both PFD0495c-GFP clones responded to PPMP in the same manner as 3D7. After a 24 hr incubation with PPMP, 3D7 parasites and both PFD0495c-GFP clones remained at the trophozoite stage rather than progressing to schizonts. When PPMP was washed out, all parasites continued the cell cycle and at day 2 matured to schizonts, which formed new rings by day 3.

**Supplemental References**

1. Bahl A, Brunk B, Crabtree J, Fraunholz MJ, Gajria B, et al. (2003) PlasmoDB: the Plasmodium genome resource. A database integrating experimental and computational data. Nucleic Acids Res 31: 212-215.

2. Lauer SA, Ghori N, Haldar K (1995) Sphingolipid synthesis as a target for chemotherapy against malaria parasites. Proc Natl Acad Sci U S A 92: 9181-9185.

3. van Ooij C, Tamez P, Bhattacharjee S, Hiller NL, Harrison T, et al. (2008) The malaria secretome: from algorithms to essential function in blood stage infection. PLoS Pathog 4: e1000084.

4. Coppel RL, Lustigman S, Murray L, Anders RF (1988) MESA is a Plasmodium falciparum phosphoprotein associated with the erythrocyte membrane skeleton. Mol Biochem Parasitol 31: 223-231.

5. Culvenor JG, Day KP, Anders RF (1991) Plasmodium falciparum ring-infected erythrocyte surface antigen is released from merozoite dense granules after erythrocyte invasion. Infect Immun 59: 1183-1187.

6. Foley M, Tilley L, Sawyer WH, Anders RF (1991) The ring-infected erythrocyte surface antigen of Plasmodium falciparum associates with spectrin in the erythrocyte membrane. Mol Biochem Parasitol 46: 137-147.
