# Supplementary figures and images for: An Erythrocyte Vesicle Protein Exported by the Malaria Parasite Promotes Tubovesicular Lipid Import from the Host Cell Surface
Source: PLoS Pathog. 2008 Aug 8;4(8):e1000118. doi: 10.1371/journal.ppat.1000118 (PMC2483944; doi:10.1371/journal.ppat.1000118)

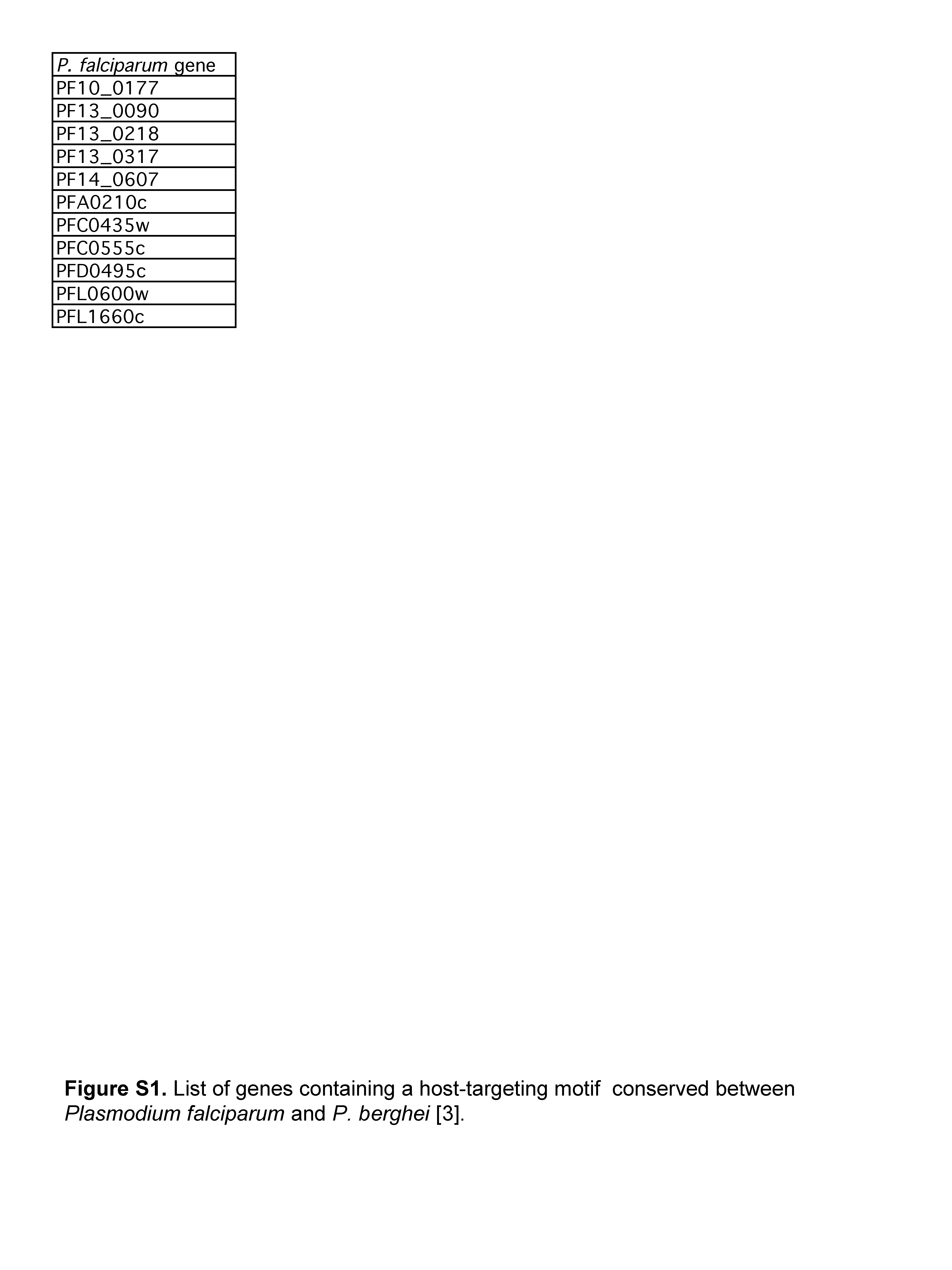

Supplement: Figure S1 — List of genes containing a host-targeting motif conserved between P. falciparum and P. berghei [3]. (0.38 MB TIF) [file ppat.1000118.s002.tif]

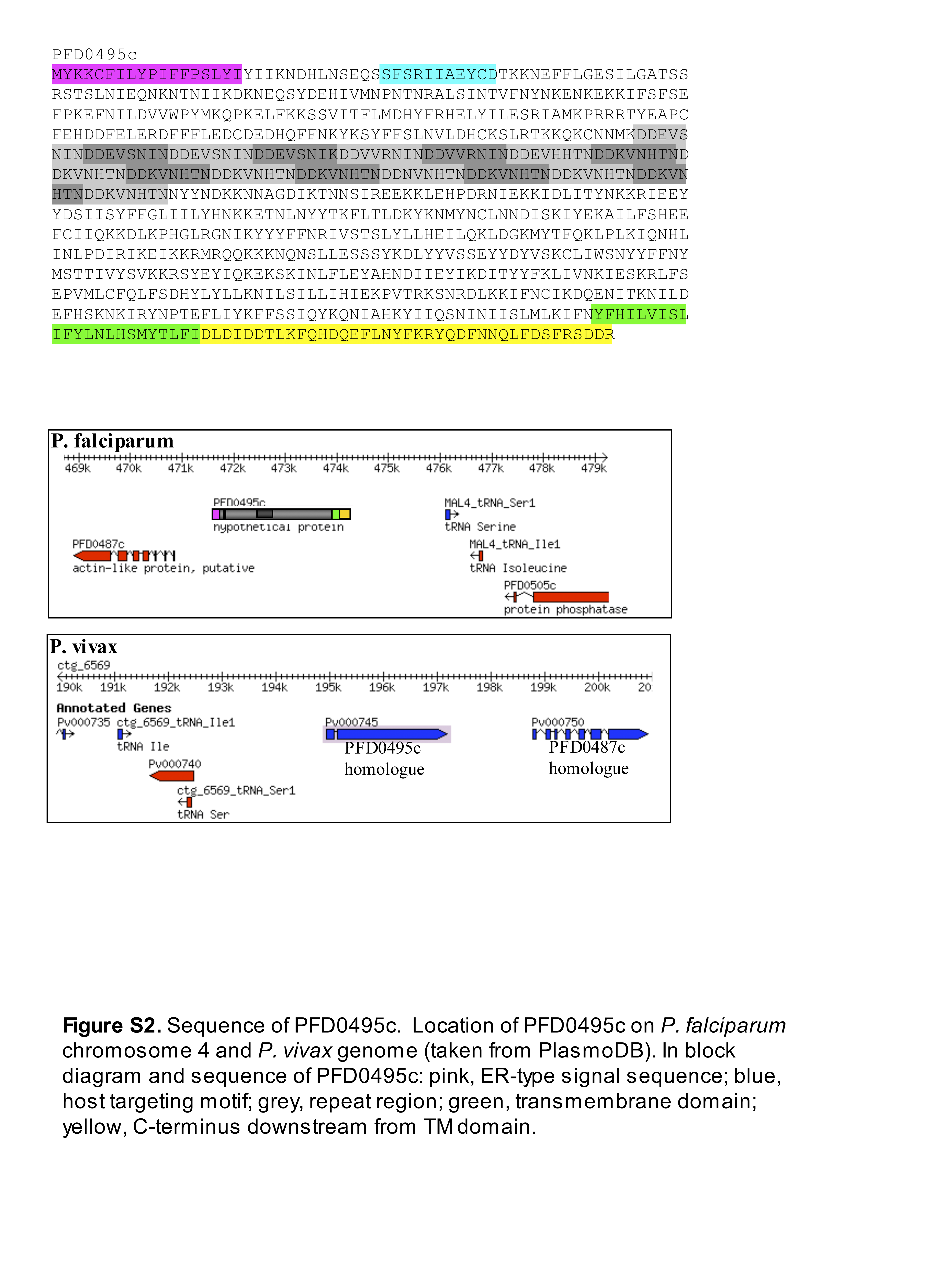

Supplement: Figure S2 — Sequence of PFD0495c. (2.13 MB TIF) [file ppat.1000118.s003.tif]

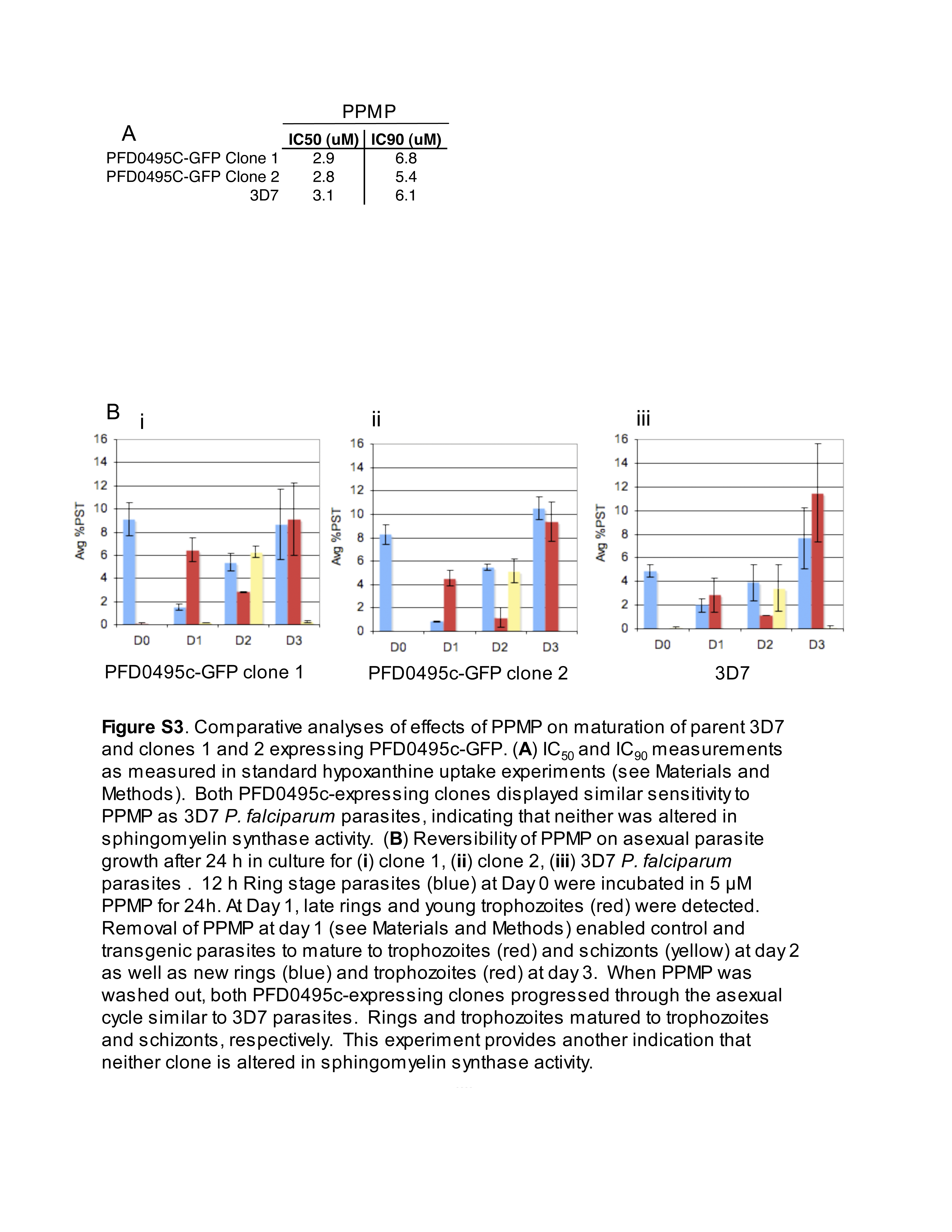

Supplement: Figure S3 — Comparative analyses of effects of PPMP on maturation of parent 3D7 and clones 1 and 2 expressing PFD0495c-GFP. (2.89 MB TIF) [file ppat.1000118.s004.tif]

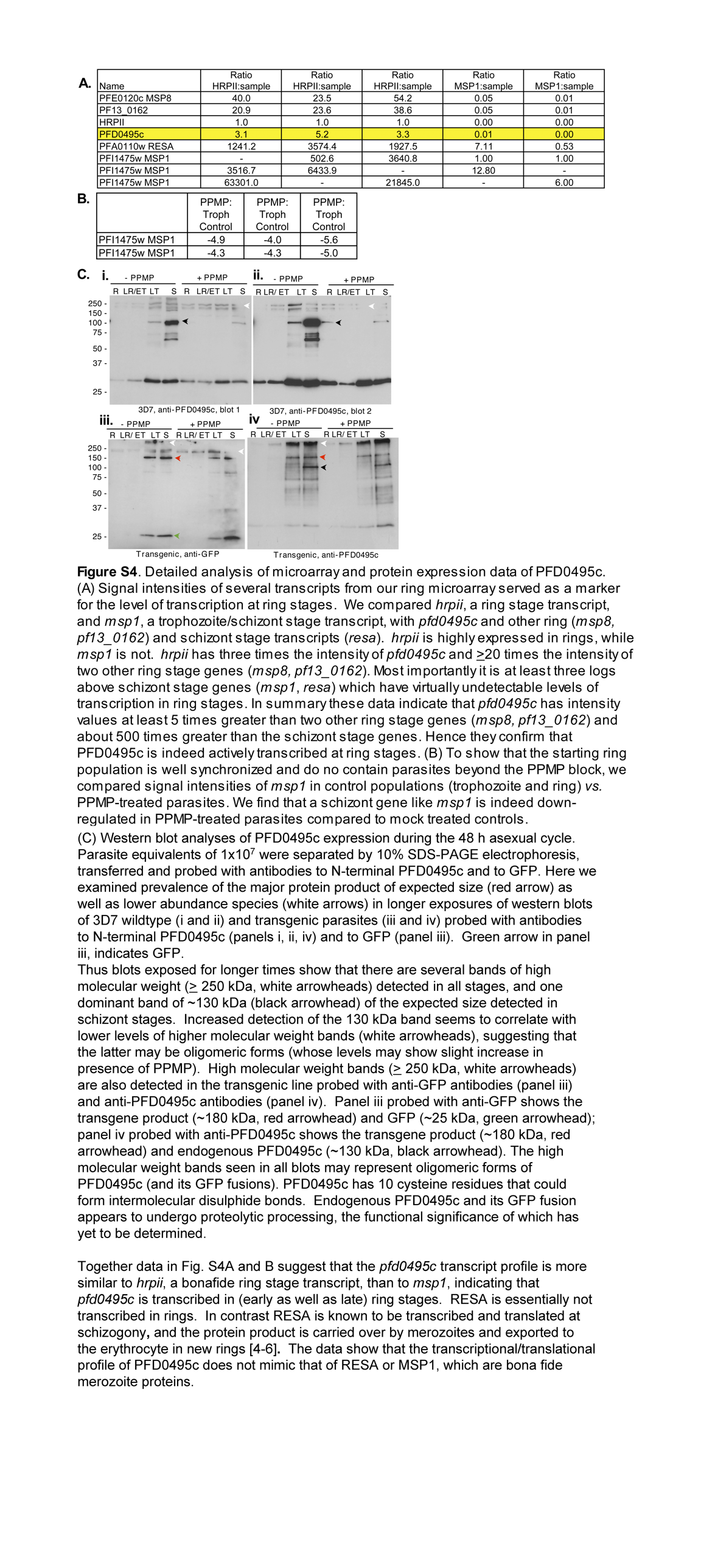

Supplement: Figure S4 — Detailed analysis of microarray and protein expression data of PFD0495c. (4.08 MB TIF) [file ppat.1000118.s005.tif]

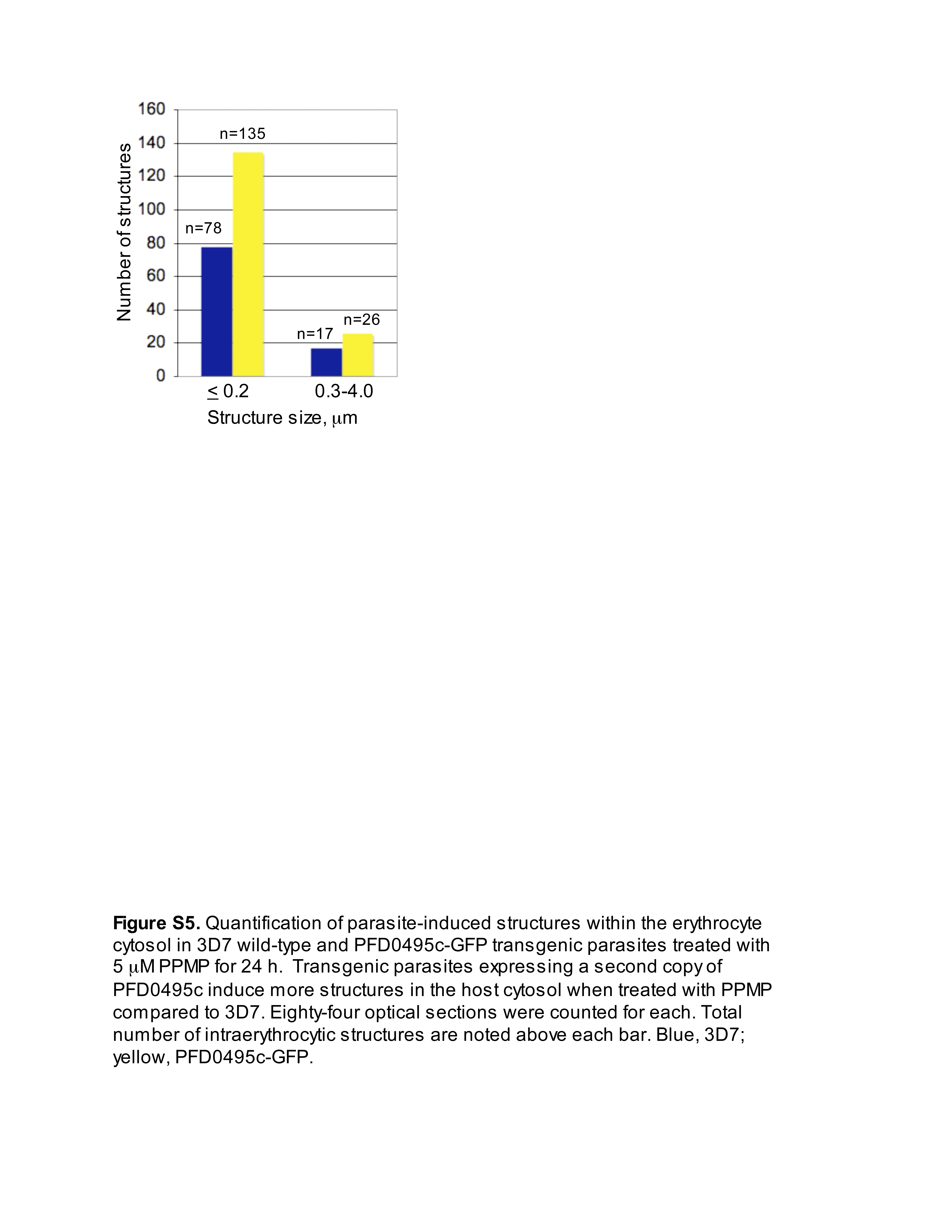

Supplement: Figure S5 — Quantification of parasite-induced structures within the erythrocyte cytosol in 3D7 wild-type and PFD0495c-GFP transgenic parasites treated with 5 µM PPMP for 24 h. (1.33 MB TIF) [file ppat.1000118.s006.tif]

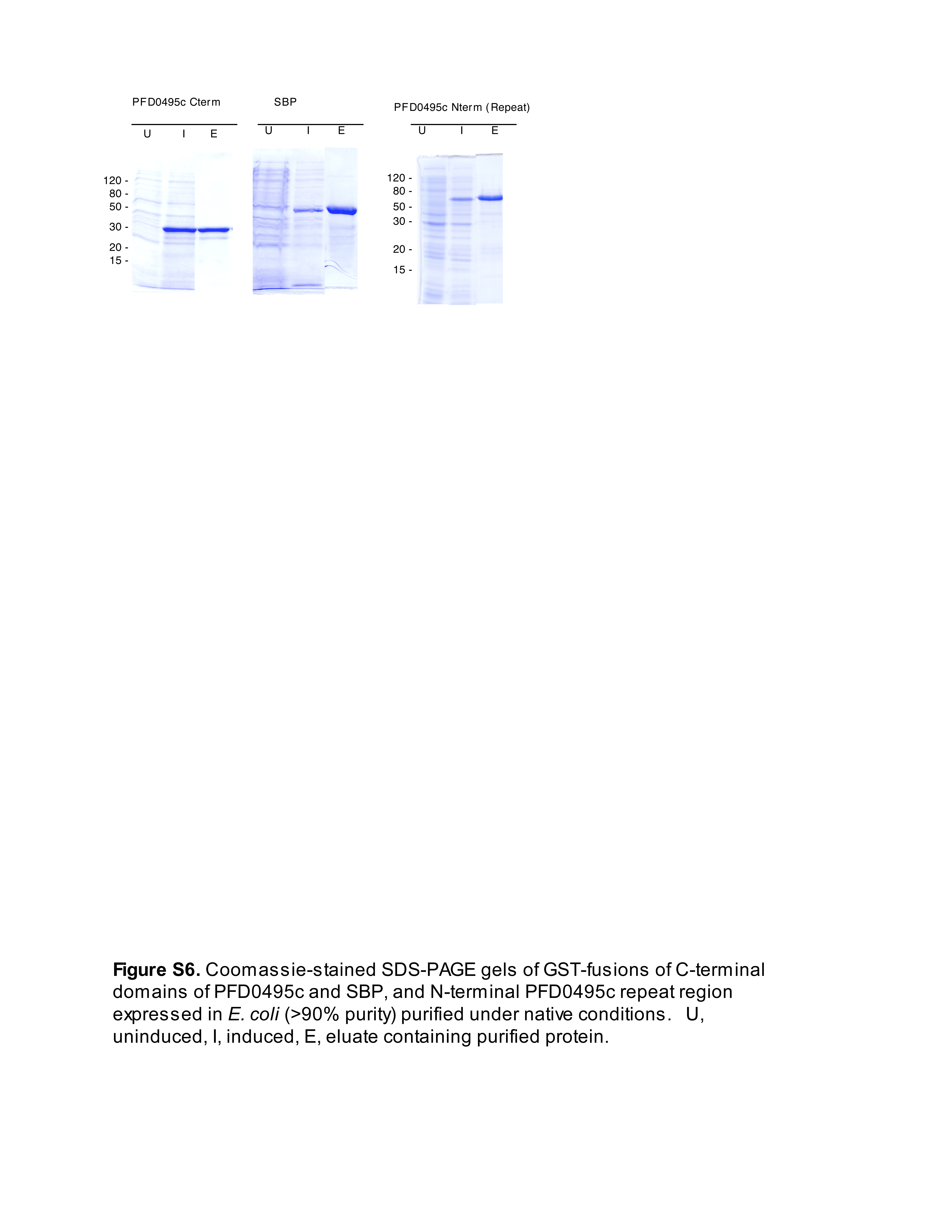

Supplement: Figure S6 — Coomassie-stained SDS-PAGE gels of GST-fusions of C-terminal domains of PFD0495c and SBP, and N-terminal PFD0495c repeat region expressed in E. coli (>90% purity) purified under native conditions. (1.46 MB TIF) [file ppat.1000118.s007.tif]

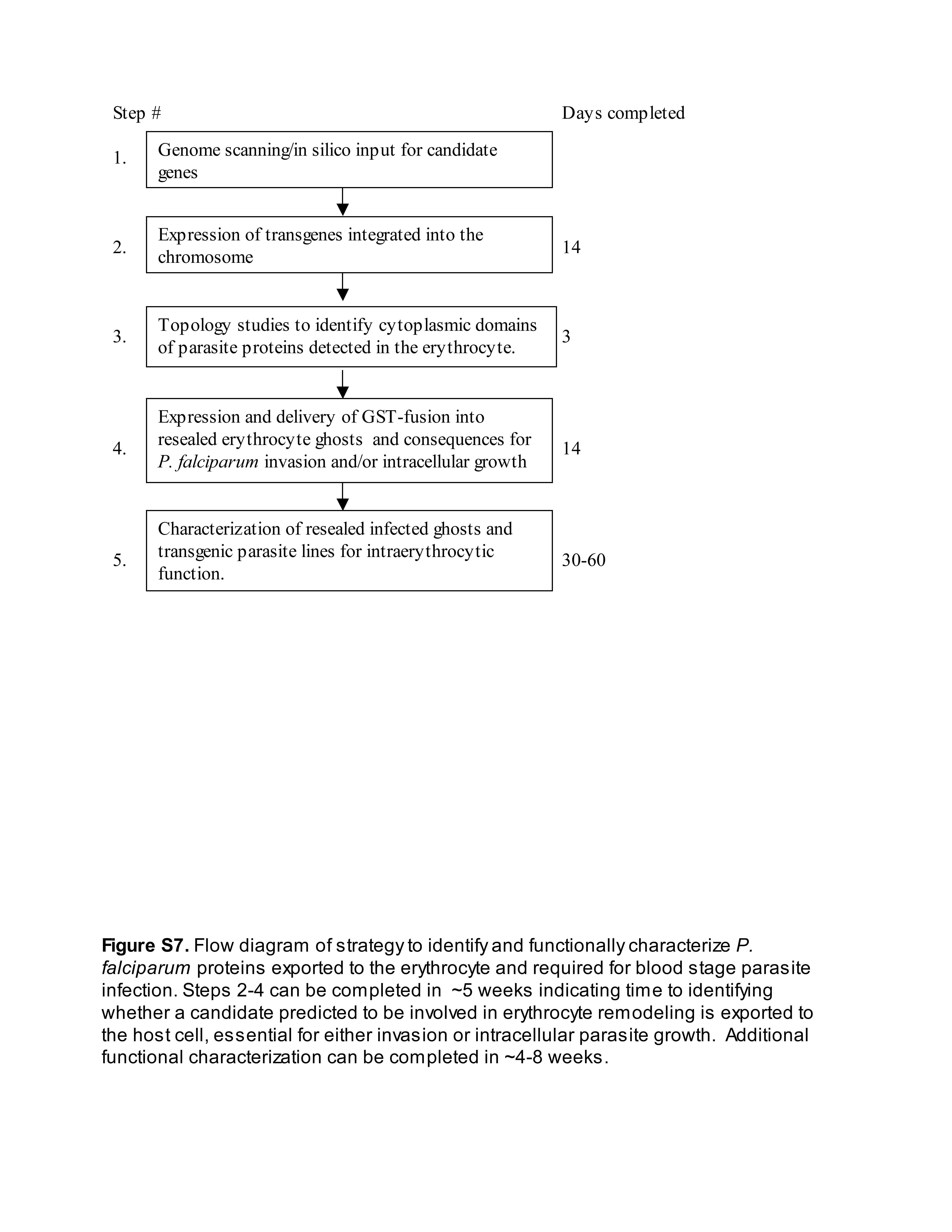

Supplement: Figure S7 — Flow diagram of strategy to identify and functionally characterize P. falciparum proteins exported to the erythrocyte and required for blood stage parasite infection. (0.99 MB TIF) [file ppat.1000118.s008.tif]
